# Acceptability of and treatment preferences for recurrent bacterial vaginosis—Topical lactic acid gel or oral metronidazole antibiotic: Qualitative findings from the VITA trial

**DOI:** 10.1371/journal.pone.0224964

**Published:** 2019-11-15

**Authors:** Jocelyn Anstey Watkins, Jonathan D. C. Ross, Sukhwinder Thandi, Clare Brittain, Joe Kai, Frances Griffiths

**Affiliations:** 1 Warwick Medical School, University of Warwick, Coventry, England, United Kingdom; 2 University Hospitals Birmingham NHS Foundation Trust, Birmingham, England, United Kingdom; 3 Nottingham Clinical Trials Unit, University of Nottingham, Nottingham, England, United Kingdom; 4 Division of Primary Care, University of Nottingham, Nottingham, England, United Kingdom; 5 Centre for Health Policy, University of the Witwatersrand, Johannesburg, South Africa; Chinese Academy of Medical Sciences and Peking Union Medical College, CHINA

## Abstract

**Background:**

Bacterial vaginosis (BV) is associated with an elevated vaginal pH and the presence of abnormal offensive discharge. It is common, often recurrent, and the most effective treatment regimen is unknown. ‘Metronidazole **V**ersus lactic ac**I**d for **T**reating bacterial v**A**ginosis’ (VITA) is a UK-based randomised controlled trial assessing clinical and cost-effectiveness of topical lactic acid gel compared to oral metronidazole antibiotic for treating second and subsequent BV episodes. Few BV trials report on women’s preferences for treatment in the context of their own experiences.

**Method:**

This qualitative study investigated the acceptability and tolerability of the two treatments. During the trial, semi-structured telephone interviews were undertaken between January—May 2018. A total of 33 women diagnosed with BV were consecutively sampled then interviewed from six sites across England. Thematic analysis was guided by the acceptability of health interventions framework. Potential causes of BV and its impact on women’s lives were explored in addition to women’s treatment preference and perceived treatment effectiveness.

**Results:**

Although women felt antibiotics treat BV effectively, and were associated with longer time periods between episodes, they generally preferred using the lactic acid gel because of ease of use, once daily application and less side-effects. Women would recommend the lactic acid gel to others for mild cases of BV but to take antibiotics when more severe. The risk of antibiotic drug resistance was a common concern. Self-help medicating or self-decision to not treat was also evident due to prior experience of poor outcomes from treatment. Triggers of BV were attributed to personal hygiene habits–soaps used to wash the vagina and sexual practices such as unprotected sex.

**Conclusion:**

Acceptability and preference for topical lactic acid gel or oral metronidazole tablets in the treatment of recurrent BV was affected by personal choice relating to *affective attitude*, *burden*, *ethicality*, *intervention coherence*, *opportunity costs*, and *self-efficacy*. These differed depending on ease of use, tolerability and past experiences, but not necessarily based on perceived drug effectiveness. Knowledge of a patient preference for topical lactic acid gel therapy despite lower perceived effectiveness may be useful for clinicians when making treatment decisions.

## Introduction

Bacterial vaginosis (BV) occurs in the lower genital tract and is often persistent with a high prevalence, varying from 20–50% amongst women of reproductive age. It has been described as ‘a common condition of unknown aetiology’ [1], and as ‘a microbiological and immunological enigma’ [2]. BV’s microbial imbalance [3] is characterised by homogenous discharge, vaginal pH greater than 4.5, positive amine test and presence of clue cells microscopically [4] but BV can also be asymptomatic. This vaginal condition may cause gynaecological and obstetric complications, and contributes to the pathogenesis of pelvic inflammatory disease [4]. Risk factors include African origin, low socioeconomic status, cigarette smoking, douching, new sexual partners and recent multiple partners [3]. The ‘polymicrobial nature of BV and its propensity for recurrence make treatment a challenge’ [5].

Treatment options are associated with a high rate of microbiological and symptomatic recurrence [6] irrespective of treatment used. At present, oral antibiotics such as metronidazole or clindamycin are regarded as first-line treatment for BV [7, 8] although they may only clear symptoms transiently. [4]. Many studies have also assessed the efficacy of lactobacilli probiotics administered intravaginally but with inconsistent results [9–11]. Another treatment option is topical lactic acid gel, available without prescription, which is promoted to restore vaginal pH balance and natural bacteria. However this is not included in current UK guidelines [12] and has not been adequately evaluated [13].

### Treatment acceptability

In addition to clinical efficacy, Devaux, Castela [14] suggest that factors to consider when selecting therapy include: patient treatment preference, the effect on the quality of life and the acceptability of treatment. Kazdin [15] defined treatment acceptability as ‘treatment that is acceptable when it is appropriate to the problem, fair, reasonable and non-intrusive to the patient’ but it must also include the degree to which the person thinks it may be effective [16]. Greater treatment acceptance is associated with higher adherence, better compliance and improved persistence [17]. To this end, acceptability has become a key consideration in the design, evaluation and implementation of healthcare interventions [18]. However, very few studies [19] have qualitatively explored acceptability and tolerability of different BV treatment options.

### Clinical trial

This paper reports on the qualitative phase of a national multi-centre trial entitled *‘Metronidazole* ***V****ersus lactic ac****I****d for* ***T****reating bacterial v****A****ginosis (VITA)*: *A randomised controlled trial to assess the clinical and cost-effectiveness of topical lactic acid gel for treating second and subsequent episodes of bacterial vaginosis’* [20, 21]. At the time of recruitment for interview, six of the 22 participating NHS sites were recruiting women to the VITA study. Participant inclusion criteria included women aged 16 years or above; with clinical diagnosis of BV based on patient-reported symptoms of unpleasant (typically fishy) odourised discharge (with or without positive microscopy from swab test according to local site practice); a history of one or more episodes of BV within the previous two years which resolved with treatment; willing to take their own vaginal samples; avoidance of vaginal douching during treatment; and avoid sexual intercourse or use effective contraception for the seven-day duration (condoms were not considered safe due to interaction with lactic acid gel). Those not eligible included women who were pregnant; breastfeeding; trying to conceive; or on other oral or topical antibiotics or antifungals. Risks to participating included possible side effects of drugs. Women were briefed that if the medication they were given did not work, further drugs could be prescribed. When seen at their local general practice (GP) surgery or genitourinary medicine (GUM) sexual and reproductive health clinic, participants were asked to take their own genital samples of vaginal discharge for microbiological analysis of BV. Women were randomised to oral metronidazole antibiotic tablets at a dose of 400mg, twice daily, 12 hours apart, for seven-days (control) or 5ml of intravaginal lactic acid gel (which is colourless and viscous) administered through an intravaginal tube and inserted into the vagina, once daily before bed, for seven-days (intervention). Participants obtained their allocated study treatment from any dispensing pharmacy as any licensed brand of metronidazole or lactic acid gel could be used. Following the initial visit, women were followed-up for resolution of their symptoms at two-weeks, three-months and six-months. Online questionnaires, optional diaries as aide-memoire and self-sampling kits were completed at home.

The aim of this predefined sub-study, as outlined in the trial protocol [21], was to use qualitative research methods to explore a diverse group of women’s experiences of the acceptability of treatments and preferences for treatment, what factors they perceived to contribute to the development and recurrence of BV, and how BV made them feel physically and emotionally.

## Methods

### Ethics statement

Ethical approval for this trial (ISRCTN14161293) was obtained from the NHS Health Research Authority, London Harrow Research Ethics Committee: 17/LO/1245 on 09/09/2017.

This study has been reported in accordance with the 21-item ‘Standards for Reporting Qualitative Research’ recommendations by O’Brien, Harris [22]. The lead author, JAW, an experienced qualitative researcher, was responsible for interview recruitment, data collection and analysis. She worked independently of the trial team and was blinded to the post-treatment results.

### Sub-study recruitment

Consecutive sampling of women who had completed the course of treatment and opted in to be approached for interview were identified. It was therefore not pre-determined how many women from each trial arm would accept an interview. Women received an email invitation for an interview from JAW and if there was no reply, two reminder emails were sent, a week apart. They were also reminded that participation was voluntary and they were free to withdraw from the interview or study at any time [21]. Interview recruitment continued until data saturation. There were no direct benefits for taking part in the trial, except a £15 Amazon gift voucher for completing a two-week post-intervention online questionnaire.

### Data collection

Semi-structured interviews were conducted over the telephone, by JAW, between January—May 2018 whilst the trial was still ongoing. Data was collected in English and interviews were scheduled at least two-weeks after completing the seven-day course of treatment. We asked women about their preferences, and the advantages and disadvantages of either treatment. Women were encouraged to talk about their recent episode and previous episodes of BV including symptoms, triggers and the impact of BV had on their lives to provide contextual background (see interview guide -Appendix 1). Women were made aware of the useful nature for us to ‘hear their voices’ in adjunct to collecting their clinical results and questionnaires at different time points. Consent was given in writing at the trial recruitment stage and again verbally at the start of each phone-call, including consent for audio-recording using an encrypted Dictaphone. Interviews were transcribed by one of the two independent transcribers hired by the University of Warwick.

### Reflexivity

Participants were told that JAW is a health science researcher (non-clinician), experienced in qualitative assessment, particularly in-depth interviews on sensitive topics. Throughout the data collection process, JAW adhered to researcher self-reflexivity around personal opinions and any experiences or preconceptions about the research. On reviewing the interviews prior to analysis, JAW emailed participants to ask for clarifications where necessary. JAW had no prior relationship with any of the participants.

### Data analysis

Data was coded by JAW in QSR International’s NVivo version 11 using thematic analysis as defined by Braun and Clarke [23]. Codes were based on interview questions and subsequent emergent themes, checked by FG via inter-coded reliability for inferences and interpretation of data. A mini-report of the first ten transcripts were analysed and shared with the wider trial team for discussion whilst data collection continued. Word spotting of descriptive adjectives assisted in summarising all contextual data describing BV symptoms. We present participant quotes labelled by their age and allocated treatment group.

### Theoretical framework

Comparative analysis followed, where we used data to relate findings to seven constructs in ‘The acceptability of health interventions framework’ by Sekhon, Cartwright [18] as the comparator. It comprises of seven multi-faceted constructs: *affective attitude* (how they feel about the intervention); *burden* (perceived effort required); *ethicality* (good fit with their value system); *intervention coherence* (how they understand the intervention); *opportunity costs* (benefits, profits, values to engage); *perceived effectiveness* (perceived as likely to achieve its purpose) and; *self-efficacy* (confidence that they can perform the behaviour required to participate). This framework fits well with our research topic on treatment acceptability, as the constructs reflect the extent to which people receiving a healthcare intervention, consider it to be appropriate, based on their anticipated or experiential cognitive and emotional responses. We consider each construct within our data from women’s’ prospective, concurrent and retrospective perspectives on BV treatments.

## Results

In this study, the biomedical treatment preferences of 33 women were exposed based on their trial participation and historical medication experience. Opinions were formed based around side-effects, perceived effectiveness, treatment outcomes, and lifestyle factors. First, we describe demographics, response rate and adherence.

### Participant demographics

We analysed interviews from the 33 women, aged 21–51 years old (average age of 32 years), from diverse ethnic backgrounds: eleven identified as Black Caribbean, nine as White, five as Black African, three as Mixed-race, two as Asian, one as Indian, one as Pakistani, and one as Chinese. Our sample was representative of most ethnicities across the six sites, whereby White and Black Caribbean, were also predominant. Our sample did not include Bangladeshi and Black Other. They were recruited from an NHS GP surgery or GUM clinic at six study sites in differing geographic locations in the West Midlands (19), the North (1) and the South East of England (13). Only six sites had been recruited onto the trial at this stage. Sexual orientation was not specifically requested, but from the interview data, we know women with both male and female partner(s) were included. Most women had experienced on average one-three episodes of BV, but 11 women had experienced BV more than three times. In our sample, 20 women had been randomised to the trial arm (oral lactic acid gel) and 13 women to the control arm (antibiotic tablets). Data saturation was not reached at the target sample of 20 women, so interviewing continued to a total of 33 participants at which time saturation had been reached.

### Response rate

Of the 52 women consecutively invited for an interview, a total of 33 women (who were two-five weeks post-treatment) completed a telephone interview, lasting between 10–45 minutes. The reasons for 19 of the women not being interviewed were: four did not answer their phones at the agreed time (even after a second attempt the next day at the same time), 13 women never replied to email invites, one woman decided not to take part in the interview, and another withdrew from the study.

### Adherence

Women generally adhered to and completed the treatment prescribed under the trial, although two forgot to take their treatment on one out of the seven-day course. Two women in the intervention-arm delayed starting treatment because of their menstruation bleed, and one went ahead using the lactic acid gel regardless of her period. All women had completed treatment before being interviewed.

The research categories are explored with related themes and described by context, treatment acceptability, perceived effectiveness and preference under Sections 1–3.

### Section 1: Context of bacterial vaginosis

#### Characteristics of the physical symptoms of bacterial vaginosis

Women described the discharge associated with BV symptoms in terms of colour, texture, smell and physical sensations using a range of adjectives (see Box 1).

**Box 1. Women’s descriptive adjectives of vaginal discharge texture, colour, smell and feel as typical symptoms of bacterial vaginosis**

**Colour of the discharge:** dark greenish, brown, yellowy, translucent, magnolia, creamy white, dark in colour, clear or whitish, pinky, cloudy

**Texture of the discharge:** solidified, crustated, mucusy, opaque, very fluid, like lotion, watery, milky, thick

**Malodour of the discharge:** rancid, very smelly, sour smell, foul kind of odour, fishy, seafood odour, potent smell, vinegary, disgusting, horrendous, stinking smell

**Physical sensation associated with the discharge:** itchiness, slight burning, irritation (symptoms worsened pre- and post-menstruation)

#### How bacterial vaginosis makes women feel and the impact on their lives

*“I just don’t feel comfortable because I feel like I always smell… There’s nothing I can do to mask it*. *Like I go through so much perfume*.*” (V33*, *aged 33*: *lactic acid gel)*

As a result of these symptoms, women expressed how these made them feel psychologically and physically. They described how it affected their mental wellbeing by making them feel depressed, increasing their anxiety and self-consciousness and for one, as a ‘life sentence’. One woman explained how she did not deal with her symptoms until breaking point.

*“It got to the point where I just couldn’t get with it anymore*. *It was affecting me at work and stuff*, *so that’s when I came in [to the GUM clinic]*. *So*, *I’ve constantly had it for a while*, *to be honest*.*” (V11*, *aged 22*: *lactic acid gel)*

BV symptoms impacted on personal relationships.

*“If I have sex with him*, *it comes*, *to the extent that I feel like I need to end the relationship*.” *(V45*, *aged 34*: *antibiotic tablets)*

Women felt ‘embarrassed’, ‘paranoid’, ‘body conscious’ and ‘self-aware’ that their vaginal discharge malodour was obvious to their partners, children, colleagues (especially if they worked closely with people: nurses, teachers, support workers) and even strangers.

*“I sat on the bus and I think people can smell me and I just stay away from people*, *you know*.” *(V24*, *aged 37*: *lactic acid gel)*

#### Episodes and recurrence of bacterial vaginosis

Several women became very emotional when describing BV as ‘a nightmare’, and how it returns and persists, even when treated. Some women described having BV all of the time or episodes as often as every two weeks.

*“Like me now*, *I’ve mastered BV; I know when it’s coming*.*” (V45*, *aged 34*: *antibiotic tablets)*

Two women said they have normalised the symptoms to the extent that they *“don’t notice a point when I don’t really have it*.*” (V23*, *aged 23*: *antibiotic tablets)*. Most women described recurrent episodes as *“really close to each other” (V15*, *aged 30*: *antibiotic tablets)* and that it feels inveterate.

*“I’ve had it for months now*. *And then I get rid of it*, *and then it comes back again*, *and then I get rid of it*, *and then it comes back again*.*” (V24*, *aged 37*: *lactic acid gel)*

Women associated recurrence with poor treatment outcomes. From their perspectives, the length of time between BV episodes was dependent on the last treatment taken:

*“After taking antibiotics*, *came back after a few months and then a few weeks after the lactic acid gel*.*” (V9*, *aged 37*: *lactic acid gel)*

Due to the frequency of recurrence, many women said they did not have spare time to repeatedly seek treatment, especially mothers.

*“Sometimes I just ignore it*, *and with work and the baby*, *I don’t really get much time*.*” (V15 aged 30*: *antibiotic tablets)*

There was a sense that women felt frustrated having tried every possible clinical intervention available to them and felt nothing worked long-term to cure their BV.

*“I was just basically at my wit’s end to try and sort this problem out… I feel like I’ve tried everything*.” *(V18*: *aged 37*, *antibiotic tablets)*

#### Perceived triggers of bacterial vaginosis

All of the women were confused about how and why they get BV recurrently. Most identified noticeable contributing triggers that impacted on recurrence. The most frequently perceived trigger that women associated with BV were sexual practices–unprotected sexual intercourse (for example, not using condoms), multiple sexual partners, and male semen post-ejaculation. All women attributed being sexually active to BV recurrence, if they were in or out of a relationship with a person(s) of the same or opposite sex and practising safe or non-safe sex.

*“I am practising*, *you know*, *quite unsafe [sexual] behaviour*, *but I’m well aware of that*.” *(V41*, *aged 33*: *lactic acid gel)*

Of those women who felt there was an association between their sexual behaviours and BV, four women believed this caused a disturbance or imbalance in their vaginal pH levels due to possible bacterial transfer.

*“I seemed to notice that if I was using tampons during my periods*, *I would like*, *without a shadow of a doubt*, *I knew I was going to have BV afterwards*.*” (V22*, *aged 28*: *antibiotic tablets)*

The use of contraceptive coils such as Intrauterine device (IUD) (copper or hormonal implant, Mirena branded), menstrual cycle and application of tampons and not changing tampons regularly or forgetting to hand wash pre-application were all viewed as contributors to the condition. Hygiene practices that were deemed to trigger BV, such as the type of soap product used by the sexual partner on their ‘intimate areas’; bathing in perfumed bubble bath water; using ‘cheap’ shower gels; cleaning with an antiseptic solution such as Dettol®; or activities such as douching were described by almost all women as detrimental to the development of BV.

*“I was told not to douche or anything like that down below*, *as it could just disrupt the natural bacteria inside*.” *(V7*, *aged 26*: *lactic acid gel)*

Further factors included: other health conditions such as endometriosis or taking prednisolone steroid medication; type of clothing material such as non-cotton underwear; type of laundry detergent such as fabric softener and wearing perfumed sanitary pads; and lifestyle choices. For example, three women suggested that their poor diets of caffeinated energy drinks and high-fat processed foods including crisps, chocolate, burgers, take-outs by *“grabbing something from either the chip shop or McDonald’s” (V15*, *aged 30*: *antibiotic tablets)* and being overweight were possible causes of BV; excessive cigarette smoking *“eight cigarettes a day” (V18*, *aged 37*: *antibiotic tablets)*; alcohol consumption particularly pinpointing the yeast in the beer when drinking *“probably eight cans and half a bottle of vodka [per day]” (V24*, *age 37*: *lactic acid gel)*, were all behaviours attributed to working shifts, multiple jobs, a lack of time and stress at work, were all mentioned.

#### Self-help: Changes in personal hygiene habits, self-help medication and home remedies

Most women had received advice about personal hygiene to prevent BV from a health professional in the past. Some had been recommended or prescribed dermatological soaps to wash with, such as Cetraben® or aqueous emollient creams. Women felt showering with a spray, using tepid tap water and a pH balance sensitive soap were preferential to bathing in fragranced soap. Soap brands used were Sienna X®, Sanex Zero%®, Aveeno®, Lush®, and Dove®. Numerous women were concerned that their body-wash products may *“still feel like it might be passing down there [vagina]” (V23*, *aged 23*: *antibiotic tablets)*. Almost half of the women said they do not use soap inside of their vagina, only water. Washing habits varied from several times per day due to religious beliefs or to remove BV discharge, or commonly twice per day or every few days to weekly (to ‘preserve the natural body oils’). Two women did not have access to a shower at their residence and used a bath or container to wash. Another said she washes her hair separately to her body, so any shampoo chemicals do not contaminate her bath water.

As a result of poor treatment effects from pharmaceutical drugs, more than half of the participants had attempted self-help medicating before, during or after being prescribed treatment for BV. This ranged from buying over-the-counter liquid lactic acid gels or waxy bullet-like pessaries either from a pharmacy or online (two brands mentioned were Canesbalance® or Balance Activ). The cost of these lactic acid gel treatments varied from £8–20 which was deemed unaffordable. The advice sought from online forums “*of women miserable because BV has ruined their life” (V8*, *aged 25*: *antibiotic tablets)* included their suggested home remedies such as steaming, washing with apple cider vinegar or inserting bio-live active natural yoghurt cultures (organisms) into the vagina.

### Section 2: Experiences and perspectives around acceptability and use of treatment for bacterial vaginosis

#### Prior experience of treatment for bacterial vaginosis

Only one woman had never experienced using the lactic acid gel before and she was randomised to the metronidazole antibiotic tablets. She was not aware that this was a treatment option. On the contrary and unusually, one woman had only ever taken the lactic acid gel to treat BV before (never before prescribed antibiotics). Another woman simultaneously sought treatment at two healthcare facilities, taking both the lactic acid gel and antibiotics at the same time. No women described previously taking alternative BV treatment types such as topical metronidazole gel or clindamycin cream.

In accordance with the acceptability of health interventions framework, we established that the following constructs aligned with our findings (Table 1).

**Table 1:** Related finding to the acceptability construct from the ‘acceptability of health interventions’ framework.

Acceptability construct	Related finding
**Self-efficacy:** *the woman’s confidence that she can perform the behaviour(s) required to participate in the intervention*	All women sampled, were by definition, willing to participate in the trial.
**(Drug) Intervention coherence:** *the extent to which the participant understands the (lactic acid gel or metronidazole antibiotic tablets) intervention and how it works*	Lactic acid gel—application once per day, at night was described as easy to apply to the site of the condition/ had a soothing effect to reduce itchiness/ was possibly messy/ application was restricted to night time/ administration was lying down.
	Metronidazole antibiotics—simple to take anywhere/ some side-effects of drugs/ concern of antibiotic resistance.
**Burden:** *the perceived amount of effort that is required to participate in the (healthcare) intervention (pros and cons)*	Lactic acid gel requires effort: comfortably lying down; application only just before bedtime at night/ abstaining from sexual intercourse for the duration of treatment (may affect intervention) (easy to buy over-the-counter in pharmacies (even within supermarkets and online) yet unaffordable.
	Metronidazole antibiotics have probable side-effects such as nausea (yet no one described this); antibiotic tablets require abstaining from alcohol (may affect intervention); obtaining prescription for antibiotic tablets requires effort (GP appointment/ drop-in service at the GUM clinic = wait time/ stigma once there/ cost of travel); course of treatment is the same length of time: seven days.
	Both treatments require more effort than self-medicating with natural home remedies, because they have to be prescribed (physically go to the appointment) or to the pharmacy.
	The burden of taking treatment lactic acid gel or antibiotic tablets put some women off = withdraw from either treatment or chance symptoms go away naturally.
	The burden of treatment was not enough for any of the women to drop out or discontinue the medication during the trial (but a few forgot on one of the treatment days).
**Affective attitude:** *how an individual feels (emotions) about the intervention*	All women were willing to give the intervention treatment a go, still willing to take it as an alternative despite the lactic acid gel not curing the symptoms of BV/or at least for only a short duration of time.
	All women were willing to try alternative treatments based on the impact BV is having on their lives/ psychosocial feelings they contended with because of the distressing symptomatology of the condition, particularly malodour and on having to take the antibiotics regularly within a period of time which made one woman feel ‘rubbish’.
**Opportunity costs:** *the extent to which benefits*, *profits or values must be given up engaging in the intervention*	For both treatments: a change in behaviour/ lifestyle is required.
	For the lactic acid gel, women may need to abstain from sexual intercourse/ delay until the menstrual cycle bleed is finished
	For metronidazole antibiotics, women must abstain from drinking alcohol.
	For both treatments, all women adhered to treatment.
**Ethicality:** *the extent to which the intervention has a good fit with an individual’s value system*	All women were prepared to do whatever it takes to get on top of the BV, ‘keep it at bay’ and not succumb to the BV and forgo value systems in pursuit of purging BV for good.
	Taking antibiotics repetitively overtime was a concern for some women.
	Taking medication treatment was not against any of the women’s principles since their lives were being governed by the BV.
	The associated side-effects of the treatment were not going to be very different from anything they had experienced in the past, mostly they had all tried one or both treatments previously.

#### Intervention treatment: Acceptability and ease of use of the lactic acid gel

As a treatment option, intravaginal lactic acid gel administered once per day, before sleeping, was overall very acceptable as it was described as ‘easy to administer/apply’ ‘without a problem’ and ‘easy to use’. Out of the 20 women randomised to the lactic acid gel, 17 described the tube applicator as comfortable to use.

*“Yeah*, *it was easy to use*, *it was fine yeah*. *Simple to follow instructions and everything yeah*.*” (V9*, *aged 37*: *lactic acid gel)*

Few problems were described in relation to lactic acid gel usage and most women had no difficulties in using the applicator or following the instructions (to lie flat on their backs at night). There was a general consensus that it was straightforward ‘to squeeze out’. Other reasons for advocating the lactic acid gel was due to its convenience:

*“Oh*, *I think it’s just kind of easier*, *it’s more convenient*. *I just don’t like taking pills*, *so I think the lactic acid gel was a better way*.*” (V15*, *aged 30*: *antibiotic tablets)*

However, one woman did worry whether the tube was empty as the angle needed to administer was ‘tricky’, causing her to struggle when squeezing out all remaining lactic acid gel. She suggested that the design of the tube could be improved by making “*the tube a bit longer” (V6*, *aged 28*: *lactic acid gel)* and she felt therefore more hygienic. Some women had prior experience of using shop-bought pessaries so were already familiar with vaginal application. Mostly everyone wore panty-liners at night during the course of treatment in case of possible seepage.

*“Obviously there is some sort of drippiness that happens a bit*. *But as it happens mostly overnight*, *it hasn’t affected me during the day at all*.*” (V8*, *aged 25*: *lactic acid gel)*

There were no side-effects associated with the lactic acid gel, except one woman said she had increased itching which she linked to the lactic acid gel rather than a symptom of BV. Another woman implied that because of the time of the day the lactic acid gel had to be applied, she forgot to use it on one occasion, as she fell asleep.

*“And sometimes I just don’t remember*, *or I’m absolutely knackered*, *and as soon as I’ve sat down on the sofa*, *I’ve dozed off*.*” (V22*, *aged 28*: *antibiotic tablets)*

She also described how she had to change her routine during the seven days of taking the lactic acid gel:

*“I mean I’ve literally got to get myself into a ritual where I’ve got to like change my bedtime routine to remember to do it and everything*.*” (V22*, *aged 28*: *antibiotic tablets)*

#### Perceived effectiveness of the lactic acid gel

The lactic acid gel was said to be fast acting, gave instant relief and improvement was felt and seen quickly. *“I noticed a very big difference*, *a very big change” (48*, *aged 39*: *lactic acid gel)* in the colour and texture of the discharge, *“it started to be see-through and sticky and look like snot again*.*” (V5*, *aged 38*: *lactic acid gel)*

All of those randomised to the lactic acid gel really wanted it to work (especially those who despised taking antibiotics) and said they would use it ‘happily’ again in the future.

*“Well I felt like*, *oh my god*, *at last something’s going to work you know*, *within seven days I’m going to be fine*. *And I can also have a drink as well*.*” (V24*, *aged 37*: *lactic acid gel)*

Several women illuminated that their symptoms cleared up during using the lactic acid gel, but the majority recalled the BV recurred within one to two weeks of completing treatment as they started presenting with the same symptoms post-treatment. They concluded that the lactic acid gel *“didn’t really work” (V37*, *aged 26*: *lactic acid gel*) and *“Yeah*, *it hasn’t worked*, *it wasn't very good at all*, *to be honest with you… it just didn’t clear it*, *it’s still there…” (V24*, *aged 37*: *lactic acid gel)*

One woman admitted being disappointed when randomised to the lactic acid gel as she had used it twice in the past and it had failed to work.

*“Whenever I had the antibiotic*, *I’d be okay for a couple of weeks*. *So*, *I wanted her to prescribe me that [the tablet]*.*” (V33*, *aged 38*: *lactic acid gel)*

#### Negative aspects of the lactic acid gel

Gel application every night was presented as a ‘faff’. It was described as messy for women menstruating and for another woman, it felt ‘irritating’ during the night. Two women described slight discomfort, cramping and abdominal pain soon after application. One participant advised that the act of coughing caused excessive lactic acid gel leakage. This caused her concern about whether she had not received the full dosage.

*“I think there was still some in there still doing good*, *but there’s some that comes out*. *So*, *I don’t know if it will affect it or not*.*” (V5*, *aged 38*: *lactic acid gel)*

Another woman said, she felt she could not drink water as usual before bedtime, in fear she would need to urinate during the night (after inserting the lactic acid gel) and was, therefore, nervous it may all come out. Two women felt the tube had too much liquid in, which caused one of them to think:

*“I felt like I’d been overloading myself with something that was foreign to my womb*. *Do you know what I mean*?*” (V33*, *aged 38*: *lactic acid gel)*

There was disagreement about whether the lactic acid gel stayed in or leaked out. Only one woman described the lactic acid gel as ‘gushing out’ when she stood up straight after application (not following the instructions). Women were advised to not engage in sexual intercourse during the lactic acid gel treatment which for some, made it a very inconvenient form of treatment.

#### Control treatment: Acceptability and ease of use of the oral metronidazole antibiotic

Overall, antibiotics were generally not acceptable to women with BV because of the frequency they had to be prescribed. However, metronidazole was considered an effective first-line prescribed treatment, particularly for ‘intense’ cases of BV. The ease of use of taking antibiotics was on all accounts called ‘easy to do’. One woman explained she was not restricted where she took the antibiotic tablets which were more practical for her.

*“You don’t have to change routine*. *People can take antibiotic tablets all the time*, *so it’s not something out of the normal*, *out of the ordinary*.*” (V3*, *aged 27*: *antibiotic tablets)*

Confidence in the metronidazole clearing up the BV was high because it was classified as an ‘antibiotic’ drug: *“Like you know it’s going to get rid of it because it’s an antibiotic*.*” (V22*, *aged 28*: *antibiotic tablets)*

Some women did not like taking antibiotics, whereas others were indifferent about taking them. The willingness to take antibiotic tablets was down to: *“I would like rid of that BV*.*” (V6*, *aged 28*: *lactic acid gel)*

#### Perceived effectiveness of metronidazole antibiotics

Some women felt antibiotics started to work within the first four days of treatment when signs of the condition started fading.

*“But obviously with the metronidazole*, *it clears up quite quickly*.*” (V35*, *aged 27*: *antibiotic tablets)*

However, women who described their symptoms disappearing after completing the course said the results were short-lived before the next episode began.

*“I’ve had metronidazole*, *it works for like two weeks and then I’ve noticed the symptoms coming back again*.” *(V38*, *aged 25*: *lactic acid gel)*

*“It improved it temporarily yeah, then it came back*.*” (V11, aged 22: lactic acid gel)*

Another woman had a similar attitude, noticing the symptoms returning albeit after a longer duration of time, sometimes up to two months, but with some of the symptoms never fully going.

*“I’m not saying it’s not working; it’s working you could say for one month*, *or you could say maximum two months*. *Now after I took the antibiotic tablets it’s working*, *but still I’ve got discharge*. *But smell has gone*.*” (V46*, *aged 42*: *antibiotic tablets)*

On the contrary, for another woman, her symptoms actually worsened throughout the seven-day treatment regimen. She felt this indicated antibiotic resistance as she had taken metronidazole several times over the years. Four women had concerns about antibiotic resistance: *“I would ask for the lactic acid gel*, *I don’t really like taking antibiotic*, *because I know your body can become like resistant to them*.*” (V11*, *aged 22*: *lactic acid gel)*

Others explained how the process of BV recurrence had become cyclical.

*“It just keeps coming back*. *I’ve been taking antibiotics before*, *and it will clear up for a little while*, *and then it will come back again*.*” (V7*, *aged 26*: *lactic acid gel)*

This was contradicted by those women who did not like the idea of taking antibiotics but did so nevertheless as they believed they worked.

*“I’m a type of person that doesn’t really like to take antibiotics anyway*. *However*, *you know*, *it was effective*.*” (V35*, *aged 27*: *antibiotic tablets)*

However, she explained this was not always accordingly to the prescribed instructions.

*“Usually I take the five antibiotic tablets in the one day*, *instead of having to take the two tablets for seven days*.” *(V35*, *aged 27*: *antibiotic tablets)*

The time frame between the symptoms going and coming back seemed to differ for each person. One woman celebrated that it had been *“over a week and a half now” (V40)* with still no signs of symptoms returning. For others, it went quickly but returned just as fast.

*“The metronidazole*, *I remember when I took it; everything was cleared up and then it just came back out of nowhere*.” *(V11*, *aged 22*: *lactic acid gel)*

#### Negative aspects of the metronidazole antibiotic

Commonly reported side-effects of metronidazole included: generally feeling ‘rubbish’, nausea, migraines and headaches, and for one woman, a dry mouth. Many women described the taste of the antibiotic tablets as ‘nasty’ or metallic.

*“Even if I halve it*, *I can’t swallow them*, *they’re quite big*. *Two*, *the taste*. *And then it causes me to be ill*, *makes me feel nauseated more*.*” (V44*, *aged 51*: *antibiotic tablets)*

Two women highlighted they *“don’t enjoy the idea of taking antibiotics a lot” (V22*, *aged 28*: *antibiotic tablets)* referring to them as ‘synthetic’: *“I prefer not to take medication if I don’t have to*.*” (V17*, *age 27*: *lactic acid gel)*. Many women had taken antibiotics for BV numerous times before:

*“The lactic acid gel*, *I prefer the idea of taking it because of the fact that you’re not pumping yourself full of antibiotics*. *Because I’m quite worried about the fact that I take*, *I need it so often*, *because I think I’ve probably had it*, *the antibiotic treatment*, *probably about eight times altogether now*.*” (V22*, *aged 28*: *antibiotic tablets)*

At least half of the women felt regularly taking antibiotics was not good for their body.

*“I hate taking antibiotics and they kind of flushed me out*.” *(V8*, *aged 25*: *antibiotic tablets)*

There was a strong sense from the women that they did not enjoy taking antibiotics for BV, despite feeling the metronidazole usually worked: *“Don’t want to take them again*.*” (V18*, *aged 37*: *antibiotic tablets)*

Another negative factor against taking metronidazole was not being able to consume alcohol for a period of time. In two instances, the participants postponed taking the treatment for a few days as it did not fit with her social life plans.

*“It’s just in the inconvenience of when I wanted to go out for a night out or have a couple of drinks with some friends*.*” (V17*, *aged 27*: *lactic acid gel)*

*“With the pills*, *I couldn’t drink alcohol*, *so that’s probably more than any other reason*. *I was like no*, *on a Friday night*.” *(V6*, *aged 28*: *lactic acid gel)*

The process of taking the antibiotics twice per day was defined as a chore: *“they’re like quite major things that impact on your life a lot*, *so taking antibiotic tablets can be a bit much*.*” (V23*, *aged 23*: *antibiotic tablets)*

This was especially true for women who did shift work, and taking antibiotic tablets was inconvenient:

*“In terms of the actual*, *the timing of my antibiotic tablets*, *because I do shift patterns*, *sometimes it’s literally like I’ll just remember oh*, *I haven’t taken it*, *and it’s a couple of hours late*.*” (V18*, *aged 37*: *antibiotic tablets)*

Also, the procedure for accessing treatment, such as antibiotics, is not immediate. Many women described the long lag-time before getting and then starting treatment. There were two cases described whereby the antibiotics were taken for BV caused a yeast infection (thrush).

### Section 3: Treatment preference for bacterial vaginosis

In this section, we describe individual preference based on the effectiveness of treatment (what the women think worked); and preference based on personal choice (what would they rather take given individual lifestyle factors/beliefs). In Table 2, we summarise the differences why one treatment was preferred to the other, according to women’s opinions depending on the severity of symptoms, the time interval between perceived recurrences, and the advantages and disadvantages of both drug treatments.

**Table 2:** Summary of women’s treatment preference based on differences between drug treatments.

Women’s treatment preference depending on:	Metronidazole Antibiotics	Lactic Acid Gel
Severity of symptoms	Severe BV	Mild BV
How often drugs can be taken	Short-term treatment	Long-term treatment
Perceived speed of cure	Quick results (all cleared up)	Quick to mid-term results
Duration between perceived recurrence	Longer durations between episodes	Shorter duration between episodes
Perceived effectiveness	Know antibiotic tablets work better = strong form of treatment	Prefers using the lactic acid gel = less intense form of treatment
Drug option	First-line treatment on the first sign of ‘severe’ symptoms	First-line treatment on the first sign of ‘mild’ symptoms
	Back-up drug (if lactic acid gel did not work the first time around)	Use in addition to antibiotic/ or straight after a course of antibiotic
Description of treatment type	Last resort treatment	Substitute treatment/ precaution/ preventative treatment

Tretament preference was almost exclusively based on the severity of the symptoms.

*“If it’s one of them [BV episode] where*, *you know*, *it doesn’t come with much of a smell*, *it’s only the discharge*, *then 100%*, *I would choose the lactic acid gel because you want it straight into the system*, *and you put it straight into the vagina*. *Whereas if it*, *I found it came with a smell*, *it came with a thick discharge*, *then definitely the tablet because within like two days of me having the tablet*, *I started*, *the smell goes*, *and the thickness of the discharge will go*.*” (V16*, *aged 30*: *antibiotic tablets)*

This is a common theme, that women would choose the lactic acid gel for perceived mild recurrences, when the BV symptoms are less severe and then switch to antibiotics for more aggressive BV. We describe why some women have a preference for treating BV with lactic acid gel over the antibiotics and the reverse.

#### Treatment preference of lactic acid gel over the antibiotic tablets

Reasons given for preference of lactic acid gel were multiple. The primary reason was availability of lactic acid gel without requiring healthcare consultation for a prescription. The lactic acid gel was deemed as “l*ess kind of invasive [than an antibiotic tablet] even though you are inserting it*.*” (V41*, *aged 33*: *lactic acid gel)*

This was related to antibiotics being regarded as ‘strong’ antibiotic tablets.

*“I don’t like taking these strong antibiotic tablets*. *It just… I don’t know I would rather insert a lactic acid gel you know*, *getting medication in my system*.*” (V10*, *aged 29*: *antibiotic tablets)*

Women seemed content with the lactic acid gel as a treatment option based on past experience with antibiotics.

*“I am*, *I’m much happier with the lactic acid gel than I was with the pills [antibiotics]*.*” (V6*, *aged 28*: *lactic acid gel)*

Women who were knowledgeable about the risks of antibiotic resistance preferred to reduce their antibiotic intake, especially since they were regular users of the same line of antibiotic therapy.

*“[Gel] is like it’s a substitute*, *because I know you can become resistant to antibiotics*.*” (V38*, *aged 25*: *lactic acid gel)*

The concern over antibiotic resistance was raised which links to why some women were keen to try another form of treatment:

*“I am quite concerned about getting resistant to using it*. *And obviously it’s quite a strong drug*, *metronidazole*, *and I’d quite like to save it in case I do need it for something quite serious in the future*. *So that’s my only sort of concern with taking that*, *you know*, *continuing to take it all the time to treat a problem that seems to never go away*.*” (V22*, *aged 28*: *antibiotic tablets)*

The lactic acid gel was easier to remember to take and applied ‘direct’ to site of the BV, making them feel like it will treat it more quickly and target the right area.

*“When you’re taking the pill*, *you don’t know if it’s aiming to the right place*.*” (V21*, *aged 30*: *lactic acid gel)*

Side effects associated with treatment were a contributing factor to their treatment choice.

*“The lactic acid gel*. *Because I remember that the antibiotics*, *I felt very tired and I really struggled to wake up in the morning*. *And yeah*, *I felt like a little bit less energy*, *you know*.” *(V31*, *aged 33*: *lactic acid gel)*

With minimal side effects, once per day administration and not having to abstain from alcohol meant it was a welcome alternative to the antibiotics.

*“The lactic acid gel*, *because then I can drink*. *Not that alcohol is the be-all and end-all of everything*.” *(V40*, *aged 41*: *antibiotic tablets)*

The lactic acid gel was described as bringing instant relief to symptoms such as itchiness. It was seen as a ‘temporary fix’ and was ‘better than nothing’. The lactic acid gel was often used as the first treatment attempted clear BV. It was also regarded as a substitute drug.

*“Well it’s difficult*, *because none of them work for me*, *so I don’t know*.*” (V10*, *aged 29*: *antibiotic tablets)*

One woman described how she intended to use the lactic acid gel preventatively straight after having sexual intercourse to act as a precautionary measure aiming to reduce the chances of BV.

*“Definitely straight after sex*, *I’m definitely going to use the lactic acid gel*. *I feel like the it will stop like things in there*.*” (V45*, *aged 34*: *antibiotic tablets)*

Another woman revealed that she had asked her the doctor to prescribe both treatments at the same time, so she could use them back-to-back in hope that by combining the drugs, this may cure the BV once and for all. The ability to buy the lactic acid gel without prescription was important to them as there is a stigma attached to going to the GUM clinic for antibiotics for BV. It also gave women the freedom to buy lactic acid gel pessaries/tubes when it suited them, rather than taking time off work for a GP appointment or visit to the GUM clinic, possibly a long wait time.

*“I have to say it’s worth it [self-medicating and paying for treatment] because whatever price it was*, *people will buy it*, *because you can’t live with BV*.*” (V38*, *aged 25*: *lactic acid gel)*

#### Treatment preference of antibiotics over the lactic acid gel

For some, the simple act of swallowing the antibiotic tablets was easier and more practical and convenient than the more physical process of inserting lactic acid gel, where they had to be at home, in a comfortable position and “*able to access that area [vagina]*.*” (V3*, *aged 27*: *antibiotic tablets)*

*“I mean I think obviously taking a tablet is easier because*, *you know*, *when you take the lactic acid gel you’ve obviously got to lie down and insert it*.*” (V41*, *age 33*: *lactic acid gel)*

Preference for the lactic acid gel was given by those who wanted to drink alcohol on weekends but who favoured antibiotics if they wanted to have sexual intercourse during treatment. The majority of women who were either randomised to metronidazole under the trial or had prior experience taking it felt *“It seems to have completely cleared up” (V22*, *aged 28*, *antibiotic tablets)*. It was implied that the antibiotics cure BV quicker:

*“I think it kind of went from like*, *I think from the next day*, *the smell kind of went… Which was really*, *really good*.*” (V15*, *aged 30*: *antibiotic tablets)*

Yet, this was dependent on the severity of the BV.

*“If it was like an extreme version*, *case of it like it was the first time I had it; I wouldn’t be against antibiotics*, *because it was so… it was affecting everything I was doing*. *But if it was more just like a kind of gentle recurrence where I thought*, *oh this is starting to be a bit different*, *then I would definitely choose the lactic acid gel*.*” (V8*, *aged 28*: *lactic acid gel)*

The lactic acid gel was described as ‘doing the trick’, however the antibiotic tablets seemed to get rid of the BV faster as they worked more quickly and was more effective with visible signs of the symptoms of BV going:

*“The lactic acid gel is still*, *you know*, *an antidote that will get rid of it*. *But I find the antibiotic tablets get rid of it quicker than the lactic acid gel*.*” (V16*, *aged 30*: *antibiotic tablets)*

The period of time between recurrence was longer when prescribed antibiotics.

*“In the future*, *in all honesty*, *I think I’d just stick to what I know*, *and that is the antibiotics*. *It does generally clear whenever I take it*.*” (V39*, *aged 27*: *lactic acid gel)*

Some felt the lactic acid gel was as effective as the antibiotic tablets and did not have any preference as the treatment outcomes were the same.

*“I can’t recall any side-effects*. *It has probably a similar result as well*.*” (V18*, *aged 37*: *antibiotic tablets)*

The lactic acid gel treatment was judged as being not necessarily the best form of treatment for curing BV but did bring instant relief.

#### Summary of treatment preferences

Overall, there was agreement that BV symptoms never actually go–they just “subside”, whatever treatment was taken.

*“The disadvantage I would say*, *that it never*, *that my*, *how would I say it*, *my symptoms never completely cleared*.” *(V38*, *aged 25*: *lactic acid gel)*

What they all have in common is none of the women has much conviction in the longevity of the biomedical treatment available on the market.

*“But on both*, *they… the BV returned after a week or two*, *on both of them*, *so I think none of them really work for me*.*” (V20*, *aged 21*: *lactic acid gel)*

*“It did yeah*, *for a couple of days*, *but then it started to come back*, *slightly*, *not as much as it has done in the past*. *But still there a bit yeah*.*” (V9*, *age 37*: *lactic acid gel)*

*“It just does not work at all… and in the seven-day course*, *no change*.*” (V24*, *aged 37*: *lactic acid gel)*

Poor treatment outcomes force many women to self-medicate as they are *‘fed up going to the clinic’ (V46*, *aged 42*: *antibiotic tablets)* as this meant time off from work, travelling there and paying for parking. Related to this, one woman felt there was a stigma associated with being seen at a GUM clinic.

*“It’s almost as though there’s a stigma attached to the fact that you’re going in there but obviously when it’s just one person over a counter [pharmacy]*, *compared to explaining your symptoms to a GP and then getting tested*.*” (V40*, *aged 41*: *antibiotic tablets)*

Another treatment preference described was to do nothing and live with the symptoms coming and going. This choice was based on poor experiences of ineffective treatments.

*“I thought right*, *let me ride out this thing and see how it goes*. *And it did reduce for a while*, *but then it came back just as quick I found*.*” (V35*, *aged 27*: *antibiotic tablets)*

Others decided to delay seeking treatment once noticing the symptoms, then watching and waiting for the BV to disappear and if not, eventually access healthcare.

*“I didn’t actually go and seek any help at that point*. *And then I waited a few months*.*” (V41*, *aged 33*: *lactic acid gel)*

#### Recommendation of treatment for bacterial vaginosis by study particpants to other women

Women were not completely convinced by either treatment as a long-term cure for vaginal recurrence. They had firm reasons for why and when they would recommend either the control or intervention treatment to someone else in their position. If it was her first episode, many endorsed the ‘milder’ lactic acid gel as a ‘top-up’ type treatment for minor cases of BV.

*“I think in my mind*, *it feels like a milder dose*, *you know*, *way of treating it rather than just going straight to the antibiotics*. *But if they were having a recurring episode*, *I would suggest the pill [antibiotic]*.*” (V33*, *aged 38*: *lactic acid gel)*

However, several women specified how their recommendation was dependent on the severity of symptoms, implying that taking antibiotics was a safer bet to clear up more moderate to severe BV.

*“I think if somebody’s genuinely got*, *you know*, *they’ve had it for a while and they’ve got a quite a bad case of BV*, *I think start with the antibiotics to make sure you 110% get rid of it*.*” (V22*, *aged 28*: *antibiotic tablets)*

Whilst, many of the women would not immediately opt for the antibiotic tablets because of their reservations described above, one woman felt she would rather be prescribed both the lactic acid gel and antibiotic tablets as a full proof method to ensure ‘it doesn’t come back’.

*“So*, *if I’m given the lactic acid gel and the antibiotics just in case it didn’t clear up*, *I’d be happy to take the lactic acid gel again*.” *(V7*, *aged 26*: *lactic acid gel)*

*“You know*, *well to me they both work*, *so I would probably advise both of them*.*” (V41*, *aged 33*: *lactic acid gel)*

Similarly, time periods between episodes were longer if antibiotics had been taken.

*“Probably the antibiotics*, *because it seemed to get rid of it for longer*.*” (V9*, *aged 37*: *lactic acid gel)*

There were women in the other camp that felt the lactic acid gel worked well enough and they would be happy to be on it for longer periods of time. Some women were not able to say which they would recommend because, for them, neither treatment had been effective:

*“Well it’s difficult*, *because none of them work for me*, *so I don’t know*.*” (V10*, *aged 29*: *antibiotic tablets)*

## Discussion

Women with recurrent BV found both treatment options: intravaginal lactic acid gel and oral metronidazole acceptable but favoured antibiotics as the most effective treatment option when the symptoms were severe, as this was perceived as having a more sustained effect. Yet in contrast, many women still preferred using the lactic acid gel, regarded as an easy, once per day application with few side-effects. Although adequate for mild cases of BV, the effectiveness of the lactic acid gel was a concern. However, the perceived rates of recurrence after using the lactic acid gel and antibiotic regimens varied between individuals.

Preference for the lactic acid gel was based on pro-factors such as, application straight to the site of the BV, offering instant soothing; concerns that frequent use of antibiotics would lead to resistance; and the availability of the lactic acid gel without prescription, even when this involved paying for it. The effort required to take the antibiotic tablets twice per day and alter social behaviours, such as drinking alcohol, played a significant role in shaping acceptability attitudes. This was often based on individual’s ideas around the practical advantages and disadvantages of each treatment, rather than whether it would actually work *(burden)*. How the BV made women feel, why they were prepared and willing to try the treatment again or for the first time *(affective attitude)*, and what they had to change as a result such as lifestyle practices or behaviours *(opportunity cost)* had a considerable influence on their decisions. Women’s treatment preferences did not always come down to effectiveness as these were often related to the woman’s value system and beliefs, and the possible consequences of treatment *(ethicality)*. All of which can be considered by the clinician prescribing the next course of treatment at the next episode.

Both treatments reduced discharge and the associated odour to some degree, sometimes within a day of starting medication. Our data suggest that women were able to take the medication according to the instructions, with only some intrusion into their daily lives *(self-efficacy)*. All women held some level of understanding about how the lactic acid gel or antibiotic tablets worked to cure the BV *(coherence)*.

All of the women described optimism for the lactic acid gel as a future alternative or replacement treatment for metronidazole. It was proposed that using both treatments back-to-back may be beneficial at keeping the BV at bay or by using the lactic acid gel as a precautionary preventative prophylaxis over prolonged periods, even when there was no clinical diagnosis of BV. The decision to not treat was also evident amongst several women who felt poor prior treatment outcomes lessened their confidence in a cure. The hope that the condition would just go away without treatment was popular. However, they also recognised that a point was often reached at which the BV became unmanageable, when treatment in whatever form was sought and led to the emotional and physical despair reported by every woman in this sub-study. Treatment preference was therefore down to personal choice, which differed depending on drug accessibility and current lifestyle, and was not necessarily focused on effectiveness. Since the trial is still ongoing (July, 2019) the comparative effectiveness of the lactic acid gel and metronidazole are still unknown to the research team.

The multifaceted nature of BV’s aetiology makes the management of acute and recurrent symptoms complicated as women’s experiences in the current study attest. Our data highlight how women with symptomatic BV have tried prescribed and self-medicated treatment options multiple times. They oscillated between which treatments they prefer, as neither lactic acid gel nor metronidazole antibiotic tablets were seen as having particularly sustained outcomes, with symptoms described as never really going away.

The characteristics of BV such as offensive smell and abnormal vaginal discharge that so impact on women’s lives are consistent with findings from other qualitative studies [1, 24]. We similarly found that the impact recurrent BV had on women’s lives goes beyond the bacterial symptomatology and leads to emotional trauma. In parallel, an increase in paranoia and reduced self-esteem affected women’s’ work and personal relationships due to the physical, emotional and social burden of living with recurrent BV [19].

There is a complex interaction of multiple components: the vaginal microbial ecosystem and the human host which is modulated by a woman’s behaviour and environment, which further complicates the overall ‘BV cause equation’ [3]. A qualitative study documenting Swedish women’s BV life experiences and management of symptoms, also found that BV influences quality of life including reduced intimacy, changes in relationships and frustration around recurrence [25]. Research into African American women with BV found that lifestyle behaviours such as douching, bathing and using vaginal cleaning agents were significantly associated with BV due to the disruption of vaginal flora [26]. Whilst women in the current study did not report these specifically, descriptions around hygiene practices as probable triggers were identified. In addition, the frequent use of self-help remedies to counteract BV was also identified in our participant group. Payne, Cromer [26] reported the potential for hormonal contraceptives to protect against BV, yet this was at odds with several women in our study, who felt there was in fact a correlation between BV and their copper coil in causing BV.

In a study by McGowan, Gomez [27], reasons for not accepting the lactic acid gel were messiness and leakage. This was seldom reported in our study, as lactic acid gel was regarded as ‘easy to use’ and non-irritating which is consistent with results from Coggins, Blanchard [28]. In our sample, women who did not prefer the lactic acid gel primarily attributed this to having to remember to insert it at night. Our results are congruent with Bilardi, Walker [19] who described how women in Australia were frustrated and dissatisfied with current treatment regimens for BV, in particular antimicrobials, leading women to take ineffective non-evidence-based therapies [24].

### Strengths and limitations

Our study findings extend the existing literature by hearing from an ethnically diverse group of women about their treatment experiences and views of BV which are valuable for practice and will help to contextualise the trial results when available. As most women had experienced various treatments, they were able to make comparisons for themselves and these prospective, concurrent and retrospective accounts gave richness to the narratives captured. However, this did mean that their experiences of different treatments sometimes merged together. They found it hard to differentiate between the most recent or previous medication (which may have been the same or different) as they were often taken so close together.

Although we aimed to interview women within two-weeks post-treatment, some interviews were delayed up to five-weeks (due to participant availability) therefore women’s recall may have become less accurate. We did not have sufficient data to undertake comparisons between demographic groups or on socioeconomic variation.

This paper has not been influenced by the quantitative results of the ongoing trial.

### Future implications

At present there is inconclusive evidence about what treatment regimen is most effective for women with recurrent BV. Our interview study would suggest, in this situation, women should be given the choice by their clinician of which treatment they prefer and/ or works best for them given their past personal experience. However, when the VITA trial results are available, this may change. If lactic acid gel is found to be the more effective treatment option, we know many women would welcome this as many prefer it. If antibiotics are found to be most effective, in many instances, women are likely to still want to use lactic acid gel because some women are hesitant to keep using antibiotics on a recurrent basis. To achieve better informed and shared decision-making about treatments, health professionals need an understanding of what women think about the use of antibiotics or topical lactic acid gel (as this may affect adherence to treatment and BV recurrence) in weighing up the pros and cons of treatments with women. Future qualitative studies are fundamental as there are still gaps related to BV, lifestyle and sexual orientation.

Approaches to tackling this common and debilitating condition need to take account of the individual’s prior experiences and retrospective acceptability by reflecting on constructs such as self-efficacy, ethicality and intervention burden, to find the best ‘treatment fit’ for each woman. Further research that considers acceptability and tolerability of BV drug options by individual preference and perceived treatment effectiveness will be necessary, if lactic acid gel is to be recommended in future treatment guidelines for clinicians to follow.

## Appendix 1. VITA study interview guide

### Introductions

#### Introduction of researcher

Reminder of study (interviewee will have received participant information sheet and signed consent form). Ask for verbal consent again on the phone and for them to confirm their full name and date of birth.

#### Confirmation that participant is happy to proceed

Explanation of what happens to the data:

- Telephone interviews will be audio recorded. The audio recordings will be stored securely. For analysis the recording will be transcribed and anonymised.

Reminder that the interviewee can stop the interview at any time.

### Opening questions

Tell me about your experiences while taking part in the VITA study?

What do you think causes your BV, if anything?

What impact does BV have on your life?

### Focusing on the treatment

Thank you. Tell me about the treatment drug you received/were randomised to for the study.

*Probes used as needed*:

*What were your initial expectations of the treatment*?
*Tell me how you used it*.
*What was good about the treatment*?
*What was not so good about the treatment*?
*Any difficulties with using it*?
*Did you have to change what you do day to day when using it*?

How satisfied are you with the treatment you received overall?

### Focusing on the modifications to make it more acceptable

Is there anything that you would add to or change about the treatment you received?

Is there anything else that you would like to say about the treatment?

Would you recommend treatment to other women with similar problems to your own?

*Probes*:

- *Explore reasons for their response*.
